# Supplementary material for: Direct assessment of possible mutations in the 23S rRNA gene encoding macrolide resistance in Chlamydia trachomatis
Source: PLoS One. 2022 May 10;17(5):e0265229. doi: 10.1371/journal.pone.0265229 (PMC9089867; doi:10.1371/journal.pone.0265229)
Supplement: Attachment — Submitted filename: Riska macrolide R Cpn 2004.pdf [file pone.0265229.s004.pdf]

ANTIMICROBIAL AGENTS AND CHEMOTHERAPY, Sept. 2004, p. 3586–3590 Vol. 48, No. 9
0066-4804/04/$08.00+0 DOI: 10.1128/AAC.48.9.3586–3590.2004
Copyright © 2004, American Society for Microbiology. All Rights Reserved.

Genetic and Culture-Based Approaches for Detecting Macrolide Resistance in *Chlamydia pneumoniae*

Paul F. Riska,[1]* Andrei Kutlin,[2] Patrick Ajiboye,[1] Arnold Cua,[1] Patricia M. Roblin,[2] and Margaret R. Hammerschlag[1,2]

Departments of Medicine[1] and Pediatrics,[2] State University of New York Downstate Medical Center, Brooklyn, New York

Received 9 January 2004/Returned for modification 8 March 2004/Accepted 19 May 2004

Three clinical *Chlamydia pneumoniae* isolates for which the MIC of azithromycin increased after treatment were investigated for genetic evidence of macrolide resistance. Attempts to induce antibiotic resistance in vitro were made. No genetic mechanism was identified for the phenotypic change in these *C. pneumoniae* isolates. No macrolide resistance was obtained in vitro.

Chlamydia pneumoniae has been linked to several chronic conditions such as atherosclerosis, chronic obstructive pulmonary disease, and asthma (4), where it is suspected of surviving in a persistent and largely asymptomatic state (13). Several long-term trials of antibiotic therapy are under way with the goal of eradicating *C. pneumoniae* and improving atherosclerotic heart disease (10). However, data on the treatment of *C. pneumoniae* infection are limited and microbiologic failure has been frequently described (12, 14, 30, 31; M. R. Hammerschlag and P. M. Roblin, Letter, Antimicrob. Agents Chemother. **44:**1409, 2000). It is unknown whether regimens used in these trials will more effectively eradicate the organism, as occurs with tetracyclines in a model of chronic *C. psittaci* infection (11), or lead to the development of antimicrobial resistance, as occurs with other microorganisms (6, 24, 26, 36).

Fortunately, drug resistance in chlamydiae is very rare (15, 21, 33, 34). However, resistance of *C. trachomatis* to quinolones and rifampin has been induced in vitro (7, 9, 22); resistance to macrolides (21) and other drugs has occasionally occurred in vivo (7, 9, 15). The veterinary pathogen *C. suis* has developed increasing resistance to tetracyclines, presumably because of chronic antibiotic exposure in animal feed (20). There is little evidence so far of development of antibiotic resistance in *C. pneumoniae* (3, 14, 31; Hammerschlag and Roblin, Letter).

In this study, we evaluated three previously reported clinical isolates of *C. pneumoniae*, from two patients with pneumonia, for which a stable fourfold increase in the MIC of azithromycin developed after treatment (30). Isolates 15-1 and 15-2 were obtained at days 0 and 18 after starting azithromycin therapy, with an increase in the MIC of azithromycin and erythromycin from 0.031 to 0.125 μg/ml (30). Isolates 72-1, 72-2, and 72-3 were obtained at 0, 9, and 37 days after starting therapy, and the MIC of azithromycin increased from 0.062 to 0.250 μg/ml for the latter isolates (30). Notably, the higher MICs still overlapped those for other wild-type *C. pneumoniae* isolates. These MICs were stable upon subculture in antibiotic-free medium. We sought genotypic evidence of this relative resistance on the basis of defined mechanisms of macrolide resistance found in other microorganisms (29). In addition, we sought to induce macrolide resistance by prolonged exposure of *C. pneumoniae* to macrolides in a standard model of acute *C. pneumoniae* infection and in an in vitro model of continuous *C. pneumoniae* infection (17–19).

Genotypic approach. Macrolide resistance usually arises by (i) mutation of the 23S rRNA at the macrolide contact site, (ii) methylation of this site by adenine *N*-methyltransferase enzymes encoded by the *erm* gene family, (iii) mutation of the L4 or L22 ribosomal protein that is expected to contact the macrolides, or (iv) a drug efflux pump mechanism (29, 38). The first mechanism occurs among organisms with single copies of the rRNA in their genome, as is the case with *C. pneumoniae* (16, 28); usually leads to high-level resistance (29); and has been demonstrated to occur in *C. trachomatis* (21). Mutations in ribosomal proteins L4 and L22 have been implicated in the development of various degrees (as little as fourfold) of macrolide resistance in *Escherichia coli*, *Bacillus subtilis*, and *Streptococcus pneumoniae* (5). Some L22 mutants of *C. trachomatis* were also associated with clinical failure (21, 37).

Sequencing of the 23S rRNA and ribosomal protein genes from our two initial and three posttreatment pneumonia isolates of *C. pneumoniae* was performed. DNA was extracted and purified with the High Pure PCR template kit (Roche). Primers for PCR (Table 1) were designed to amplify two predicted macrolide resistance regions of domain V of the 23S rRNA and the genes encoding the L4 and L22 proteins. The relevant 23S rRNA region was identified by homology to the sequence of *Mycobacterium avium* (24) and to bp 2057 to 2059 and 2610 to 2611 of the *E. coli* gene (38) and encompassed bp 2026 to 2028 and 2580 to 2581 of the *C. pneumoniae* AR39 23S rRNA gene (28). The L4 and L22 primers spanned the chlamydial equivalent of codons 63 to 74 of L4 (37) and the C-terminal region of L22 (23), which have mutations in some macrolide-resistant strains of *S. pneumoniae*.

A systematic comparison by BLAST (http://www.ncbi.nlm.nih.gov/sutils/genom_table.cgi; last accessed 23 April 2004) of other proteins involved in macrolide resistance (reviewed in reference 29) revealed significant homologies of three *Staphy-*

* Corresponding author. Mailing address: Box 56, 450 Clarkson Ave., Brooklyn, NY 11203. Phone: (718) 270-4181. Fax: (718) 270-4123. E-mail: riska@att.net.

Downloaded from https://journals.asm.org/journal/aac on 05 December 2021 by 138.5.240.55.

TABLE 1. PCR oligodeoxynucleotide primers used in this study

Primer name	Sequence[a]	Primer position[b]	PCR product size (bp)
23S-F	5′-TCATAGTCTCCTGGCTATCC-3′	2103	175
23S-R	5′-GACCTGCACGAATGGTGTAA-3′	1928	
23Sb-F	5′-ATTCCGGTCCTCTCGTACTA-3′	2642	212
23Sb-R	5′-CGCCACCAAGAGTTCATATC-3′	2431	
L4-F	5′-CGCTCAACAAGTTCCTGCAAAG-3′	26	434
L4-R	5′-AATGCCCGTCAGGGATGTTTAG-3′	459	
L22-F	5′-CTTTCTGACCCATGATCC-3′	−28	405
L22-R	5′-GCCATCCTGTGAAGAAAG-3′	386	
Erm-1	5′-GA(A/G)ATIGGIIIIGGIAA(A/G)GGICA-3′		530
Erm-2	5′-AA(C/T)TG(A/G)TT(C/T)TTIGT(A/G)AA-3′		
KsgA-F	5′-ATTACGCAAGCGCTACGGCTAT-3′	−93	930
KsgA-R	5′-CCTTTAACCCGCTTGCATCT-3′	833 + 3	
YgeD5-F	5′-GTGGTCTGACAAGACATGTA-3′	−181	1,193
YgeD5-R	5′-CATGGAGAGGCACTTGATA-3′	1011	
YgeD3-F	5′-TTTGCTTGTTCGATACTATT-3′	940	819
YgeD3-R	5′-TAAGAACGATTTACGGTATCTTA-3′	1698 + 60	
Yjjk-3	5′-GCTCCTCGTGTTAGATAC-3′	1593 + 19	939
Yjjk-3int	5′-ACGACACGATCATTATCTAC-3′	673	
Yjjk-5	5′-GGTCTATACGCAAGTCTC-3′	−121	957
Yjjk-5int	5′-ACTTGACTCGCTCGTGAT-3′	835	

[a] T, thymidine; C, cytosine; A, adenosine; G, guanine; I, inosine; (X/Y), nucleotides X and Y present in equal parts.
[b] Base position relative to the translational start site of the gene cited in the primer name; a plus sign indicates base pairs situated after the 3′ end of the gene.

lococcus aureus proteins to predicted gene products in the *C. pneumoniae* genome. ErmA (accession no. BAB42746, 243 amino acids [aa]), a 23S rRNA ribosomal methylase, has 24% identity and 42% similarity over its first 215 aa to the *C. pneumoniae* dimethyladenosine transferase known as the kasugamycin resistance gene product (KsgA, accession no. Q9Z6K0, 565 aa). Second, the macrolide efflux gene product (MefA, accession no. AAL58635, 405 aa) has homology to a *C. pneumoniae* putative efflux protein (YgeD, NP_224932.1, 565 aa) within its N-terminal one-third. Remarkably, the *S. aureus* NorA multidrug efflux protein (accession no. BAB56857, 388 aa) (27), although not homologous to MefA, also has 23% identity and 43% similarity to YgeD, in a central region of the protein spanning 195 aa. Finally, the virginiamycin resistance gene product (VgaB, accession no. U82085, 552 aa), an ATP-binding efflux pump, has 32% identity and 53% homology over its C-terminal two-thirds with the chlamydial YjjK gene product (accession no. NP_224231, 530 aa), a putative ATP-binding transporter. It is worth noting that the macrolide-streptogramin resistance gene products MsrC of *Enterococcus faecalis* (accession no. NP_815134) and MsrA of *Staphylococcus epidermidis* (accession no. P23212) are homologous to both VgaB and YjjK in a similar region. By contrast, no significant homologies could be found in the chlamydia genomes to macrolide hydrolases (VgbA, VgbB), esterases (EreA, EreB), phosphorylases (MphA), transferases (LnuA) (see reference 29 for a review), or other multidrug efflux proteins (AcrAB, TolC, LmrA, NorM, Mmr, EmrB) or their regulators (AcrR, BltR, BmrR, Mta) (see reference 27 for a review).

Primers were designed to amplify the chlamydial gene homologues and upstream region (Table 1). PCR was performed with the Expand PCR kit (Roche), 25 μM primers, denaturation at 95°C for 5 min, and then 30 cycles of 94°C for 30 s, 60°C (50°C for *yjjK*) for 30 s, and 72°C for 30 s, followed by extension at 72°C for 7 min. The PCR fragments obtained were resolved on a 1% agarose gel, spin column purified, and then sequenced in both directions with the individual PCR primers. Sequence results were reviewed manually and compared with the BLAST2 program (http://www.ncbi.nlm.nih.gov/BLAST/bl2seq/bl2.html) to the four reference *C. pneumoniae* sequences in the GenBank database (accession numbers AE001363, AE002161, BA000008, and NC_005043). Additionally, PCR with degenerate primers previously shown to amplify the family of *erm* genes from diverse species (1) was performed with a macrolide-resistant *S. aureus* clinical isolate as a positive control.

Single bands were obtained from each of the 23S rRNA, L4, L22, *ksgA*, *ygeD*, and *yjjK* PCRs, and except for that of *ksgA*, each DNA sequence was identical to that of the four reference strains in the GenBank database. The *ksgA* gene has two known polymorphisms, with K108T and R161K mutations found only in the TW-183 reference isolate among the four sequenced isolates, as well as in each of our clinical isolates. No *erm* gene product was identified from the *C. pneumoniae* isolates with degenerate primers, while a single 530-bp band was obtained from the positive control. Thus, no mechanism was found to account for the shift in the MIC for these isolates.

Since the function or expression level of the chlamydial Erm,

Downloaded from https://journals.asm.org/journal/aac on 05 December 2021 by 138.5.240.55.

MefA/NorA, and Vga/Msr homologues is unknown, a potential role in low-level macrolide resistance cannot be excluded by sequencing alone. It is possible that overexpressed KsgA can methylate the 23S rRNA target of macrolides, assuming the role of *erm* proteins. KsgA normally methylates adenine residues on the 16S RNA, increasing the activity of the non-macrolide antimicrobial kasugamycin. Mutations in *ksgA* have rarely been implicated in macrolide resistance in a mutant of *E. coli* with multiple other ribosomal protein mutations (32). Conversely, the presence of an *erm* gene in *E. coli* increases kasugamycin sensitivity, presumably by cross-methylating the 16S RNA (35). By contrast, the functions, natural substrates, and potential for macrolide efflux of the proteins YgeD and YjjK are unknown. YjjK is an ATP-hydrolyzing transporter (27), while YgeD is a member of the major facilitator superfamily of membrane transporters that use the transmembrane proton gradient (27) and most homologous to acyltransferases. Members of the major facilitator superfamily are often regulated by other proteins, which may have escaped detection by homology. Thus, our future work will consider the expression of these protein homologues.

Arguments used to explain heterotypic resistance might also be applicable to our isolates. Specifically, within the complex life cycle of the chlamydiae, there are metabolically inert forms such as the elementary body that are likely to be unaffected by antibiotics, as well as intermediate and aberrant forms that may be preferentially induced under antibiotic pressure (2, 9). There is no light microscopy evidence, however, that these atypical forms are increased in clinical isolates for which the MICs are higher (data not shown). We might also have missed small subpopulations of stably resistant isolates against a background of susceptible ones. Newer methods that would be more sensitive than direct sequencing for these minor populations and more adaptable to the screening of larger numbers of isolates, such as heteroduplex analysis (25) or single-strand conformational polymorphisms (5), are being considered for future studies.

Phenotypic methods. Induction of macrolide resistance in an acute *C. pneumoniae* infection model was attempted by inoculating HEp-2 cells with *C. pneumoniae* TW-183 (ATCC VR 2282) at a high multiplicity of infection (5×10^7 inclusion-forming units/ml). The medium was then replaced with one with one-half or one-fourth of the MICs of azithromycin (0.12 or 0.06 μg/ml), clarithromycin (0.03 or 0.016 μg/ml), and levofloxacin (0.5 or 0.25 μg/ml) and incubated for 72 h at 35°C. Six blind passages over 21 days were performed in the presence of the same antimicrobials, after which plates were fixed and stained with antichlamydial antibodies specific for lipopolysaccharide (Pathfinder; Bio-Rad, Redmond, Wash.).

Induction of antimicrobial resistance in a persistent-infection model was attempted by replacing the growth medium in continuous *C. pneumoniae* CM-1 (ATCC VR-1360) cultures (18) with medium with or without 0.03 μg of azithromycin per ml, 0.008 μg of clarithromycin per ml, and 0.125 μg of levofloxacin per ml (each at one-eighth of the MIC). Cultures were incubated for 30 days, and the medium was changed and fresh antibiotics were added every 3 days. Samples were obtained on days 0, 6, 12, 18, 24, and 30 for direct detection of chlamydial inclusions after fixing of adherent cells and staining with antichlamydial antibody. Infectious-titer assays were carried out on day 30 as previously described (30).

We were unable to induce stable antimicrobial resistance in *C. pneumoniae* after repeated passages in subinhibitory concentrations of azithromycin, clarithromycin, and levofloxacin with conventional acute culture and continuously infected cells. Surprisingly, even concentrations of antimicrobials below the MIC, when applied for longer durations than in typical MIC determinations, proved inhibitory to *C. pneumoniae* development in the acute-infection model. At least for the macrolides, this effect could be confirmed in the continuous-infection model, which showed several indicators of drug activity.

First, low-dose azithromycin or clarithromycin treatment of *C. pneumoniae* in continuously infected HEp-2 cells prevented host cell lysis, despite an initial increase in the inclusion number. It is this early increase in inclusions that could be interpreted as nonefficacy of the drug in a short-term MIC assay. Only at later time points did these more typical inclusions disappear almost completely.

Second, the inclusions that were seen became progressively irregular in the low-dose-macrolide-treated host cells and not like the "atypical inclusions" described in the chronic-infection model without antibiotics (18). The inclusions stain less intensely, appear amoeboid, and seem to have a disrupted border. Similar inclusions have also been noted after azithromycin treatment of an established *C. trachomatis* infection (9). We noted that much of the chlamydial lipopolysaccharide detected by antibody appears to be extracellular, either as infectious elementary bodies or, more likely, as debris, as suggested by Wyrick et al. (39).

Lastly, in our study, stable viable chlamydiae could not be recovered after the macrolide regimens, despite an initial successful round of replication after removal of the drug. However, it is possible that some residual macrolide was being transferred in these assays, leading to continued inhibition of chlamydial development. The effects of macrolide levels below the MIC on the continuously infected cultures are similar to those of high levels (at least 16 times the MIC) which are comparable to those found in the lung after typical dosing regimens (17). The lower limit of macrolide effects on chlamydial growth in our continuous-infection model are being explored. Others have found that azithromycin treatment of established *C. trachomatis* infection (at the minimal bactericidal concentration) led to loss of infectivity (9), while quinolone treatment of *C. trachomatis* seemed to maintain viable chlamydiae after drug withdrawal (8). Thus, the disruption of chlamydial development by macrolides seems to be more complete than that which occurs with quinolones and perhaps is less likely to allow opportunities for resistance to develop. This result may occur because of intraepithelial cell concentration of the macrolide drugs, leading to effectively higher drug exposure. Understanding the minimally effective macrolide concentration in our chronic-infection model may allow us to select more appropriate drug dosing to induce resistance or avoid it in the future.

Our results also suggest that macrolides should be quite potent against *C. pneumoniae* in vivo, if levels were maintained long enough. However, these results may not apply to monocytes (12), and the documented failures of eradication may be

Downloaded from https://journals.asm.org/journal/aac on 05 December 2021 by 138.5.240.55.

due to *C. pneumoniae* in such sanctuary sites, which are unable to concentrate macrolides.

Repeated passage in subinhibitory concentrations of various antibiotics including macrolides reproducibly induces resistance in other bacteria (6, 26). Our attempts to select for resistant chlamydia strains are in contrast to those of Dessus-Babus et al. (7), who induced high-level resistance to ofloxacin and sparfloxacin in *C. trachomatis* after four passages in subinhibitory concentrations (one-half of the MIC) of the drugs. Our results are more similar to those of Morrissey et al. (22), who also were able to induce resistance to ofloxacin and ciprofloxacin in *C. trachomatis*, but not in *C. pneumoniae* after more than 30 passages in subinhibitory concentrations of the quinolones. As with the macrolides in the chronic infection in our study, viable forms of *C. pneumoniae* seemed to occur after passage in sparfloxacin but could not be propagated. Clearly, *C. pneumoniae* is different from *C. trachomatis* and other bacteria.

C. pneumoniae appears not to develop high-level antibiotic resistance very readily and to be susceptible to even low levels of macrolides (one-eighth of the MIC) in our in vitro chronic-infection model, which is reassuring. However, more subtle changes in macrolide susceptibility have been demonstrated in clinical isolates, and these need to be further defined, as they may be the first steps toward genuine high-level resistance in *C. pneumoniae*. Finally, most published studies of treatment of *C. pneumoniae* infection have relied on serology for diagnosis, rather than culture, and this makes it impossible to detect or monitor for the emergence of resistance. Recognizing drug resistance if and when it occurs is especially important in view of the ongoing studies of prolonged antibiotic treatment for prevention of cardiac morbidity (10), which could inadvertently both induce resistance and be compromised by it.

P.F.R. was supported by NIH grant KO8 AI01628. This work was largely funded through a Dean's Initiative Research Grant from the State University of New York—Downstate Medical Center.

Valuable assistance was provided by Kurt Cullamar, Michelle Then, and Stephan Kohlhoff.

REFERENCES

1. **Arthur, M., C. Molinas, and C. Mabilat.** 1993. PCR detection of *erm* erythromycin resistance genes by using degenerate oligonucleotide primers, p. 534–538. *In* D. Persing, T. Smith, F. Tenover, and T. J. White (ed.), Diagnostic molecular microbiology: principles and applications. American Society for Microbiology, Washington, D.C.
2. **Beatty, W. L., R. P. Morrison, and G. I. Byrne.** 1994. Persistent chlamydiae: from cell culture to a paradigm for chlamydial pathogenesis. Microbiol. Rev. **58:**686–699.
3. **Block, S., J. Hedrick, M. R. Hammerschlag, G. H. Cassell, and J. C. Craft.** 1995. *Mycoplasma pneumoniae* and *Chlamydia pneumoniae* in pediatric community-acquired pneumonia: comparative efficacy and safety of clarithromycin vs. erythromycin ethylsuccinate. Pediatr. Infect. Dis. J. **14:**471–477.
4. **Boman, J., and M. R. Hammerschlag.** 2002. *Chlamydia pneumoniae* and atherosclerosis: critical assessment of diagnostic methods and relevance to treatment studies. Clin. Microbiol. Rev. **15:**1–20.
5. **Canu, A., B. Malbruny, M. Coquemont, T. A. Davies, P. C. Appelbaum, and R. Leclercq.** 2002. Diversity of ribosomal mutations conferring resistance to macrolides, clindamycin, streptogramin, and telithromycin in *Streptococcus pneumoniae*. Antimicrob. Agents Chemother. **46:**125–131.
6. **Davies, T. A., G. A. Pankuch, B. E. Dewasse, M. R. Jacobs, and P. C. Appelbaum.** 1999. In vitro development of resistance to five quinolones and amoxicillin-clavulanate in *Streptococcus pneumoniae*. Antimicrob. Agents Chemother. **43:**1177–1182.
7. **Dessus-Babus, S., C. M. Bebear, A. Charron, C. Bebear, and B. de Barbeyrac.** 1998. Sequencing of gyrase and topoisomerase IV quinolone resistance-determining regions of *Chlamydia trachomatis* and characterization of quinolone-resistant mutants obtained in vitro. Antimicrob. Agents Chemother. **42:**2474–2481.
8. **Dreses-Werringloer, U., I. Padubrin, B. Jurgens-Saathoff, A. P. Hudson, H. Zeidler, and L. Kohler.** 2000. Persistence of *Chlamydia trachomatis* is induced by ciprofloxacin and ofloxacin in vitro. Antimicrob. Agents Chemother. **44:**3288–3297.
9. **Dreses-Werringloer, U., I. Padubrin, H. Zeidler, and L. Kohler.** 2001. Effects of azithromycin and rifampin on *Chlamydia trachomatis* infection in vitro. Antimicrob. Agents Chemother. **45:**3001–3008.
10. **Gabay, M. P., and R. Jain.** 2002. Role of antibiotics for the prevention of cardiovascular disease. Ann. Pharmacother. **36:**1629–1636.
11. **Galasso, G., and G. Manire.** 1961. Effect of antiserum and antibiotics on persistent infection of HeLa cells with meningopneumonitis virus. J. Immunol. **86:**382–385.
12. **Gieffers, J., H. Fullgraf, J. Jahn, M. Klinger, K. Dalhoff, H. A. Katus, W. Solbach, and M. Maass.** 2001. *Chlamydia pneumoniae* infection in circulating human monocytes is refractory to antibiotic treatment. Circulation **103:**351–356.
13. **Hammerschlag, M. R.** 2002. The intracellular life of chlamydiae. Semin. Pediatr. Infect. Dis. **13:**239–248.
14. **Hammerschlag, M. R., and P. M. Roblin.** 2000. Microbiologic efficacy of moxifloxacin for the treatment of community-acquired pneumonia due to *Chlamydia pneumoniae*. Int. J. Antimicrob. Agents **15:**149–152.
15. **Jones, R. B., P. B. Van der Pol, D. H. Martin, and M. K. Shepard.** 1990. Partial characterization of *Chlamydia trachomatis* isolates resistant to multiple antibiotics. J. Infect. Dis. **162:**1309–1315.
16. **Kalman, S., W. Mitchell, R. Marathe, C. Lammel, J. Fan, R. W. Hyman, L. Olinger, J. Grimwood, R. W. Davis, and R. S. Stephens.** 1999. Comparative genomes of *Chlamydia pneumoniae* and *C. trachomatis*. Nat. Genet. **21:**385–389.
17. **Kutlin, A., C. Flegg, D. Stenzel, T. Reznik, P. M. Roblin, S. Mathews, P. Timms, and M. R. Hammerschlag.** 2001. Ultrastructural study of *Chlamydia pneumoniae* in a continuous-infection model. J. Clin. Microbiol. **39:**3721–3723.
18. **Kutlin, A., P. M. Roblin, and M. R. Hammerschlag.** 1999. In vitro activities of azithromycin and ofloxacin against *Chlamydia pneumoniae* in a continuous-infection model. Antimicrob. Agents Chemother. **43:**2268–2272.
19. **Kutlin, A., P. M. Roblin, and M. R. Hammerschlag.** 2002. Effect of prolonged treatment with azithromycin, clarithromycin, or levofloxacin on *Chlamydia pneumoniae* in a continuous-infection model. Antimicrob. Agents Chemother. **46:**409–412.
20. **Lenart, J., A. A. Andersen, and D. D. Rockey.** 2001. Growth and development of tetracycline-resistant *Chlamydia suis*. Antimicrob. Agents Chemother. **45:**2198–2203.
21. **Misyurina, O. Y., E. V. Chipitsyna, Y. P. Finashutina, V. N. Lazarev, T. A. Akopian, A. M. Savicheva, and V. M. Govorun.** 2004. Mutations in a 23S rRNA gene of *Chlamydia trachomatis* associated with resistance to macrolides. Antimicrob. Agents Chemother. **48:**1347–1349.
22. **Morrissey, I., H. Salman, S. Bakker, D. Farrell, C. M. Bebear, and G. Ridgway.** 2002. Serial passage of Chlamydia spp. in sub-inhibitory fluoroquinolone concentrations. J. Antimicrob. Chemother. **49:**757–761.
23. **Musher, D. M., M. E. Dowell, V. D. Shortridge, R. K. Flamm, J. H. Jorgensen, P. Le Magueres, and K. L. Krause.** 2002. Emergence of macrolide resistance during treatment of pneumococcal pneumonia. N. Engl. J. Med. **346:**630–631.
24. **Nash, K. A., and C. B. Inderlied.** 1995. Genetic basis of macrolide resistance in *Mycobacterium avium* isolated from patients with disseminated disease. Antimicrob. Agents Chemother. **39:**2625–2630.
25. **Nash, K. A., and C. B. Inderlied.** 1996. Rapid detection of mutations associated with macrolide resistance in *Mycobacterium avium* complex. Antimicrob. Agents Chemother. **40:**1748–1750.
26. **Pankuch, G. A., S. A. Jueneman, T. A. Davies, M. R. Jacobs, and P. C. Appelbaum.** 1998. In vitro selection of resistance to four beta-lactams and azithromycin in *Streptococcus pneumoniae*. Antimicrob. Agents Chemother. **42:**2914–2918.
27. **Putman, M., H. van Veen, and W. Konings.** 2000. Molecular properties of bacterial multidrug transporters. Microbiol. Mol. Biol. Rev. **64:**672–693.
28. **Read, T. D., R. C. Brunham, C. Shen, S. R. Gill, J. F. Heidelberg, O. White, E. K. Hickey, J. Peterson, T. Utterback, K. Berry, S. Bass, K. Linher, J. Weidman, H. Khouri, B. Craven, C. Bowman, R. Dodson, M. Gwinn, W. Nelson, R. DeBoy, J. Kolonay, G. McClarty, S. L. Salzberg, J. Eisen, and C. M. Fraser.** 2000. Genome sequences of *Chlamydia trachomatis* MoPn and *Chlamydia pneumoniae* AR39. Nucleic Acids Res. **28:**1397–1406.
29. **Roberts, M. C., J. Sutcliffe, P. Courvalin, L. B. Jensen, J. Rood, and H. Seppala.** 1999. Nomenclature for macrolide and macrolide-lincosamide-streptogramin B resistance determinants. Antimicrob. Agents Chemother. **43:**2823–2830.
30. **Roblin, P. M., and M. R. Hammerschlag.** 1998. Microbiologic efficacy of azithromycin and susceptibilities to azithromycin of isolates of *Chlamydia pneumoniae* from adults and children with community-acquired pneumonia. Antimicrob. Agents Chemother. **42:**194–196.
31. **Roblin, P. M., G. Montalban, and M. R. Hammerschlag.** 1994. Susceptibilities to clarithromycin and erythromycin of isolates of *Chlamydia pneu-*

Downloaded from https://journals.asm.org/journal/aac on 05 December 2021 by 138.5.240.55.

moniae from children with pneumonia. Antimicrob. Agents Chemother. **38:**1588–1589.
32. **Saltzman, L., and D. Apirion.** 1976. Binding of erythromycin to the 50S ribosomal subunit is affected by alterations in the 30S ribosomal subunit. Mol. Gen. Genet. **143:**301–306.
33. **Somani, J., V. B. Bhullar, K. A. Workowski, C. E. Farshy, and C. M. Black.** 2000. Multiple drug-resistant *Chlamydia trachomatis* associated with clinical treatment failure. J. Infect. Dis. **181:**1421–1427.
34. **Suchland, R. J., W. M. Geisler, and W. E. Stamm.** 2003. Methodologies and cell lines used for antimicrobial susceptibility testing of *Chlamydia* spp. Antimicrob. Agents Chemother. **47:**636–642.
35. **Suvorov, A. N., B. van Gemen, and P. H. van Knippenberg.** 1988. Increased kasugamycin sensitivity in *Escherichia coli* caused by the presence of an inducible erythromycin resistance (erm) gene of *Streptococcus pyogenes*. Mol. Gen. Genet. **215:**152–155.
36. **Tait-Kamradt, A., T. Davies, P. C. Appelbaum, F. Depardieu, P. Courvalin, J. Petitpas, L. Wondrack, A. Walker, M. R. Jacobs, and J. Sutcliffe.** 2000. Two new mechanisms of macrolide resistance in clinical strains of *Streptococcus pneumoniae* from Eastern Europe and North America. Antimicrob. Agents Chemother. **44:**3395–3401.
37. **Tait-Kamradt, A., T. Davies, M. Cronan, M. R. Jacobs, P. C. Appelbaum, and J. Sutcliffe.** 2000. Mutations in 23S rRNA and ribosomal protein L4 account for resistance in pneumococcal strains selected in vitro by macrolide passage. Antimicrob. Agents Chemother. **44:**2118–2125.
38. **Vester, B., and S. Douthwaite.** 2001. Macrolide resistance conferred by base substitutions in 23S rRNA. Antimicrob. Agents Chemother. **45:**1–12.
39. **Wyrick, P. B., S. T. Knight, T. R. Paul, R. G. Rank, and C. S. Barbier.** 1999. Persistent chlamydial envelope antigens in antibiotic-exposed infected cells trigger neutrophil chemotaxis. J. Infect. Dis. **179:**954–966.

Downloaded from https://journals.asm.org/journal/aac on 05 December 2021 by 138.5.240.55.
