# Supplementary material for: Direct assessment of possible mutations in the 23S rRNA gene encoding macrolide resistance in Chlamydia trachomatis
Source: PLoS One. 2022 May 10;17(5):e0265229. doi: 10.1371/journal.pone.0265229 (PMC9089867; doi:10.1371/journal.pone.0265229)
Supplement: Attachment — Submitted filename: Rebuttal.docx [file pone.0265229.s005.docx]

January 21th, 2022
Dear Editors,

We thank the editors and reviewers for their input and comments on the manuscript and we have edited the manuscript to address their concerns. In the next section, we’ll provide a point-by-point response to the comments.

1. *Please address all concerns of reviewer 1*.
Reviewer #1: *I would change the title to “Direct assessment of possible mutations in the 23S rRNA gene encoding macrolide resistance in Chlamydia trachomatis”, as they didn't find any mutations. These mutations also confer resistance to other macrolides including erythromycin*.

We agree with reviewer 1 and have changed the title of our manuscript, as suggested.


2. *Under Clinical Evaluation in Methods, please briefly describe the methods that were used to determine CT positive clinical samples, CT quantitative load (including how IFU/mL was determined) and Ct typing, even if these methods are in reference 2, as this will facilitate a more complete understanding of the data*.

We have added the nucleic acid amplification test used to screen for *Chlamydia trachomatis* infections, we have described the CT quantification as reported by reference 2, and added the methods used to perform sequence typing.

3. *Although Figure S1 is reproduced from reference 2, please include as a main figure with appropriate permissions*.

We have included ‘Figure S1’ as a main figure in the manuscript (Fig 2). We have emailed PLOS ONE for permission regarding reuse of this figure. In the reply we received 19.01.2022, it was stated that the content was published under an open access license (CC-BY). The original creators are credited in the Figure’s caption.

4. *Please change ‘golden’ to gold; change ‘to best of our knowledge’ to ‘to the best of our knowledge’*.

We have edited this in the current manuscript.

5. *Please ensure that your manuscript meets PLOS ONE's style requirements, including those for file naming*.

We have edited the manuscript according to PLOS ONE’s style requirements, including file naming.

6. *Please review your reference list to ensure that it is complete and correct. If you have cited papers that have been retracted, please include the rationale for doing so in the manuscript text, or remove these references and replace them with relevant current references. Any changes to the reference list should be mentioned in the rebuttal letter that accompanies your revised manuscript. If you need to cite a retracted article, indicate the article’s retracted status in the References list and also include a citation and full reference for the retraction notice*.

The reference list is complete and correct. One reference has been added, as described below (point 10).

7. *In your Data Availability statement, you have not specified where the minimal data set underlying the results described in your manuscript can be found. PLOS defines a study's minimal data set as the underlying data used to reach the conclusions drawn in the manuscript and any additional data required to replicate the reported study findings in their entirety. All PLOS journals require that the minimal data set be made fully available*.

We have provided all accession numbers required to reproduce Fig 2 in the supplementary file called ‘S1_File’.
We also provided the accession numbers of the *Chlamydia suis* and *Chlamydia muridarum* 23S gene used to find discriminatory regions. These accession numbers have been added to ‘S2_File’. All 23S sequences generated in the current study have been submitted to GenBank. We have been provided accession numbers for these sequences (OM320821 to OM320875). Our sequences are currently being examined and processed by the GenBank annotation staff to ensure that it is free of errors or problems.

8. *We note that you have included the phrase “data not shown” in your manuscript. Unfortunately, this does not meet our data sharing requirements. PLOS does not permit references to inaccessible data. We require that authors provide all relevant data within the paper, Supporting Information files, or in an acceptable, public repository. Please add a citation to support this phrase or upload the data that corresponds with these findings to a stable repository (such as Figshare or Dryad) and provide and URLs, DOIs, or accession numbers that may be used to access these data. Or, if the data are not a core part of the research being presented in your study, we ask that you remove the phrase that refers to these data*.

We have removed the phrase “data not shown” and added the discriminatory regions under the results section (technical evaluation). The *Chlamydia suis* and *Chlamydia muridarum* sequences used to evaluate these discriminatory regions, have been added to the supplementary ‘S2_File’.

9. *While revising your submission, please upload your figure files to the Preflight Analysis and Conversion Engine (PACE) digital diagnostic tool*.

All 3 main figures have been uploaded to the PACE tool, and LZW compressed.

10. Attachment ‘Riska macrolide R Cpn 2004.pdf’

Although we were uncertain about the aim of the added attachment (‘Riska macrolide R Cpn 2004.pdf’), we were reminded of this very interesting paper with indeed many similarities with our work on CT. We have added this reference in the introduction, 3^rd^ paragraph.

We look forward to hearing from you regarding our submission. We would be glad to respond to any further questions and comments that you may have.

On behalf of all authors,

Julius van Niekerk
